# Tubular Carcinoma of the Breast: Advantages and Limitations of Breast Tomosynthesis

**DOI:** 10.1155/2016/3906195

**Published:** 2016-12-26

**Authors:** Filipa Vilaverde, Ana Rocha, Alcinda Reis

**Affiliations:** ^1^Serviço de Imagiologia, Centro Hospitalar de Entre o Douro e Vouga, Santa Maria da Feira, Portugal; ^2^Servicio de Radiología, Hospital Povisa, Vigo, Spain

## Abstract

Tubular carcinoma of the breast is a rare variant of invasive ductal carcinoma. We report a case of 42-year-old asymptomatic female with a histopathological proven multifocal tubular carcinoma, studied by mammography, Tomosynthesis, Ultrasound, and Magnetic Resonance. Herein, we discuss the advantages and limitations of Tomosynthesis, an emerging imaging technique, in this particular case.

## 1. Introduction

Mammography, the accepted technique for breast cancer screening, presents a global sensitivity of about 85%, reducing breast cancer deaths by 15 to 30% [1, 2]. Mammographic screening false negatives vary from 6 to 46% between series and are much more common in dense breasts. This is mainly related to the fact that a three-dimensional structure—the breast—is being studied with a two-dimensional image with subsequent tissue overlap [1, 3].

Digital Breast Tomosynthesis (DBT) is a new tool that can be expected to partially obviate this problem by reducing or eliminating tissue overlap. DBT technology enables the acquisition of a three-dimensional volume of thin-section data, and images are reconstructed in conventional orientations by using reconstruction algorithms similar to those used in computed tomography (CT). DBT is being progressively implemented in breast imaging clinics, as early clinical data have shown that it improves the accuracy of screening and diagnostic breast imaging, addressing some of the long-standing limitations of conventional mammography [4–6].

However, as with any new technique, several issues must be considered when implementing DBT into daily practice, and some limitations of the technique must be recognized.

This case illustrates some potential advantages and limitations of DBT.

## 2. Clinical Case

A 42-year-old asymptomatic female presented to our institution for routine screening breast study. The patient had family history of breast cancer (her mother at 45 years). Physical examination was normal.

**Figure 1:**
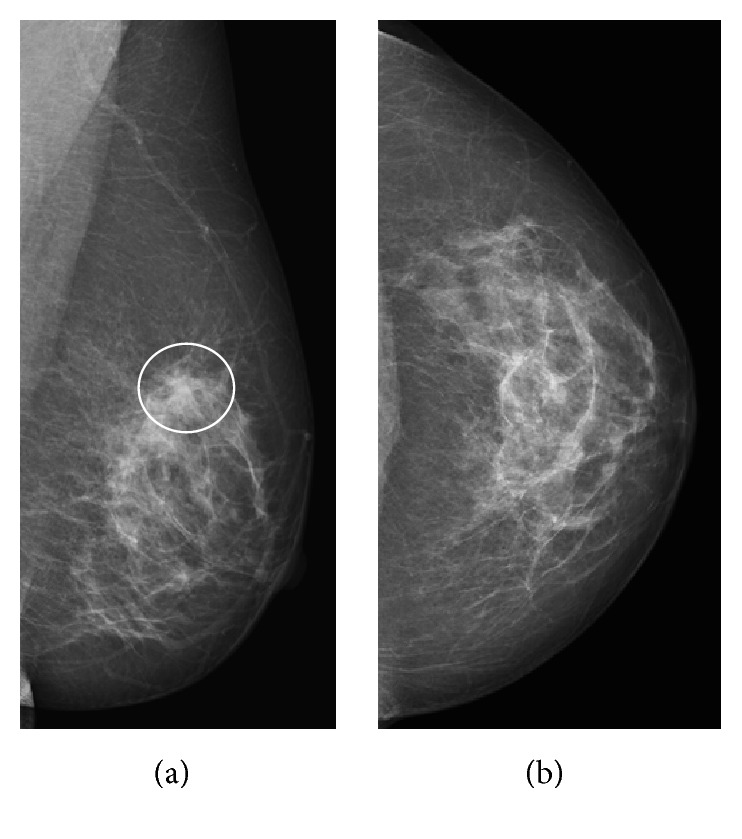
Left breast mammography, mediolateral oblique (a) and craniocaudal views (b). There is an architectural distortion in the upper quadrants of the left breast (white circle), not clearly seen in the craniocaudal view: Rocha A, Servicio de Radiologia, Hospital Povisa, Vigo, Spain.

**Figure 2:**
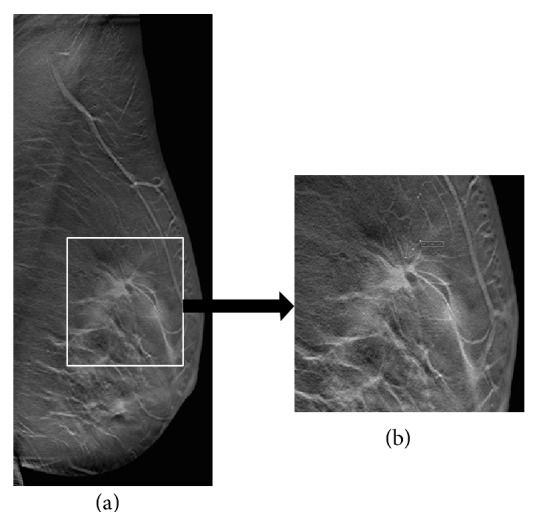
Left mediolateral oblique Tomosynthesis image section (a), with pathologic area close-up view (b). There was improved architectural distortion visualization further localized to the upper outer quadrant. Close-up view shows a small central mass with large spicules and focal retraction along the margin of the tissue: Rocha A, Servicio de Radiologia, Hospital Povisa, Vigo, Spain.

**Figure 3:**
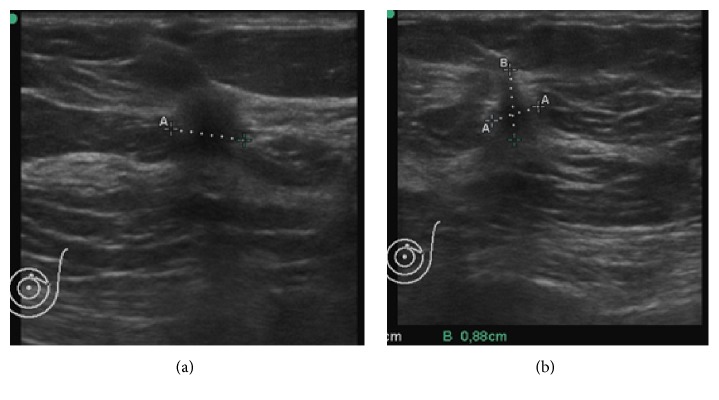
Ultrasound, transverse (a) and longitudinal (b) sections. A 10 mm solid nodule was shown, with irregular and ill-defined margins, taller rather than wider shaped, with a hyperechoic rim and posterior acoustic shadowing: Rocha A, Servicio de Radiologia, Hospital Povisa, Vigo, Spain.

Mammography showed an architectural distortion in the upper quadrants of the left breast, only clearly seen in the mediolateral oblique view (Figure 1). DBT—routinely performed at the patient's hospital—localized the lesion to the upper outer quadrant and further characterized it as a small irregular mass with long spicules (Figure 2). A targeted Ultrasound (US) showed a 10 mm solid nodule, with posterior acoustic shadowing (Figure 3). A tru-cut biopsy guided by US was performed, revealing a tubular carcinoma.

**Figure 4:**
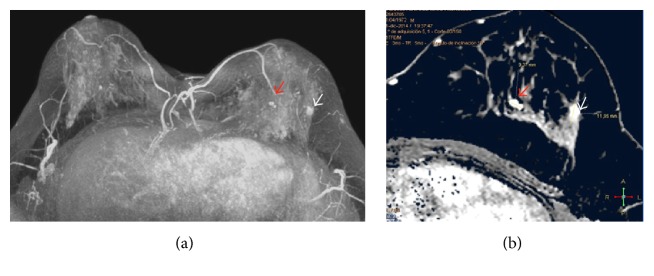
Breast Magnetic Resonance T1-weighted images, after gadolinium administration and axial maximum intensity projection (MIP) reformations. In addition to the previously biopsied lesion in the upper outer quadrant, there was another nodule 35 mm apart, in the upper inner quadrant. It measured 8 mm and was also submitted to US-guided biopsy: Rocha A, Servicio de Radiologia, Hospital Povisa, Vigo, Spain.

Breast Magnetic Resonance Imaging (MRI) was done to evaluate the local extension and showed, apart from this nodule, an additional one 8 mm in the upper inner quadrant, with the same imaging findings at MRI and US, and at a distance of 35 mm from the former (Figure 4). It was biopsied at second-look US, and the histologic diagnosis was also tubular carcinoma.

## 3. Discussion

Tubular carcinoma of the breast is a well-differentiated type of invasive ductal carcinoma that forms neoplastic tubules mimicking breast ductules and accounting for about 1% of all breast carcinomas [7]. It may contain other histologic elements, but an excess of 75% tubular elements is usually required for the diagnosis of tubular carcinoma [1]. Lesions are multifocal or multicentric in around 15% of cases [1]. Tubular carcinoma typically occurs in a younger population than the more common Invasive Ductal Carcinoma Not Otherwise Specified (IDC-NOS) [1]. The prognosis is excellent with survival of 97% at 10 years [8].

The lesion is usually very small (<1 cm) and nonpalpable, often first detected on screening mammography [8]. Some cases may be mammographically occult [9].

Tubular carcinoma presents on mammography as an architectural distortion or as an irregularly shaped mass with spiculated margins, with or without calcifications. The appearance mimics IDC-NOS. The spicules are often longer than the central mass [8, 9]. On US, the appearance also mimics IDC-NOS, manifesting as a hypoechoic solid mass with ill-defined margins and posterior acoustic shadowing. MRI shows characteristics of a malignant tumor, more commonly an irregular mass with a type 3 enhancement curve (rapid initial rise, followed by a drop-off with time/washout in the delayed phase) [8, 9]. Although DBT did not allow a specific diagnosis in this particular case, it was helpful for lesion localization seen on only one mammographic view, therefore obviating the need for additional projections. By scrolling through the image slices, a slider tool displays where the current image slice is positioned anatomically in a medial or lateral location on the mediolateral view and the superoinferior location on the craniocaudal view. Also, DBT improved diagnosis confidence in a malignant lesion, showing a central mass with large spicules, which was not featured on mammography. This is in accordance with known advantages of DBT: improved conspicuity and characterization of lesions due to reduced obscuration by overlapping breast tissue [5, 6]. Described false negatives of DBT are mainly related to areas of dense tissue without fat planes that blur tumor margins, particularly in the smaller ones [4]. Probably, an area of denser tissue was responsible for the inconspicuous second tumor.

Tubular carcinoma should be considered in the differential diagnosis of a small spiculated mass, especially if it has long spicules [10].

Our patient was submitted to partial mastectomy and the final diagnosis was compatible with multifocal tubular carcinoma with some foci of IDC-NOS, low grade.

## 4. Conclusion

DBT improves radiologist's diagnostic confidence allowing differentiation between fibroglandular tissue overlap and a true lesion, without the need of additional mammographic acquisitions. As tubular carcinoma frequently presents with an architectural distortion or as a small mass, it represents a potential tumor benefiting from DBT.
